# Effect of methyl jasmonate and GA3 on canola (*Brassica napus* L.) growth, antioxidants activity, and nutrient concentration cultivated in salt-affected soils

**DOI:** 10.1186/s12870-024-05074-9

**Published:** 2024-05-09

**Authors:** Subhan Danish, Sundas Sana, Muhammad Baqir Hussain, Khadim Dawar, Hesham S. Almoallim, Mohammad Javed Ansari, Misbah Hareem, Rahul Datta

**Affiliations:** 1 Department of Soil Science, Faculty of Agricultural Sciences & Technology, Bahauddin Zakariya University, Multan, Punjab Pakistan; 2 Department of Botany, The Islamia University of Bahawalpur, Sub-campus Rahim Yar Khan, Rahim Yar Khan, Pakistan; 3 Department of Soil and Environmental Sciences, Muhammad Nawaz Shareef University of Agriculture, Multan, Punjab Pakistan; 4 Department of Soil and Environmental Science, The University of Agriculture, Peshawar, Pakistan; 5 Department of Oral and Maxillofacial Surgery, College of Dentistry, King Saud University, PO Box-60169, Riyadh, 11545 Saudi Arabia; 6 Department of Botany, Hindu College Moradabad (Mahatma Jyotiba Phule Rohilkhand University Bareilly), Moradabad, India; 7 Department of Environmental Sciences, Woman University Multan, Multan, Punjab Pakistan; 8 Department of Geology and Pedology, Faculty of Forestry and Wood Technology, Mendel University in Brno, Zemedelska 1, Brno, 61300 Czech Republic

**Keywords:** Antioxidants, Growth hormones, Growth attributes, Nutrients concentration, Salinity stress

## Abstract

Salinity stress is a significant challenge in agricultural production. When soil contains high salts, it can adversely affect plant growth and productivity due to the high concentration of soluble salts in the soil water. To overcome this issue, foliar applications of methyl jasmonate (MJ) and gibberellic acid (GA3) can be productive amendments. Both can potentially improve the plant’s growth attributes and flowering, which are imperative in improving growth and yield. However, limited literature is available on their combined use in canola to mitigate salinity stress. That’s why the current study investigates the impact of different levels of MJ (at concentrations of 0.8, 1.6, and 3.2 mM MJ) and GA3 (0GA3 and 5 mg/L GA3) on canola cultivated in salt-affected soils. Applying all the treatments in four replicates. Results indicate that the application of 0.8 mM MJ with 5 mg/L GA3 significantly enhances shoot length (23.29%), shoot dry weight (24.77%), number of leaves per plant (24.93%), number of flowering branches (26.11%), chlorophyll a (31.44%), chlorophyll b (20.28%) and total chlorophyll (27.66%) and shoot total soluble carbohydrates (22.53%) over control. Treatment with 0.8 mM MJ and 5 mg/L GA3 resulted in a decrease in shoot proline (48.17%), MDA (81.41%), SOD (50.59%), POD (14.81%) while increase in N (10.38%), P (15.22%), and K (8.05%) compared to control in canola under salinity stress. In conclusion, 0.8 mM MJ + 5 mg/L GA3 can improve canola growth under salinity stress. More investigations are recommended at the field level to declare 0.8 mM MJ + 5 mg/L GA3 as the best amendment for alleviating salinity stress in different crops.

## Introduction

Global challenges like climate change and urbanization highlight the need for plants to thrive in adverse conditions [1, 2]. Factors such as marginal land use and unsustainable irrigation increase global salinity, threatening crop yields by disrupting plant physiological processes [3–5]. Salinity stress leads to elevated osmotic pressure and salt toxicity, impacting seed germination, growth, and reproductive behavior [6, 7]. Furthermore, it inhibits the growth of microorganisms, which is essential for plant development and the cycling of nutrients [8–10]. Plants experience cellular damage due to reactive oxygen species (ROS) produced in response to salinity stress [11]. Plants have developed antioxidant defense systems comprising non-enzymatic antioxidants like ascorbate enzymatic antioxidants and superoxide dismutase to mitigate ROS damage and preserve cellular homeostasis [11, 12]. Recognizing these mechanisms is essential for developing ways to improve crop resilience to salinized surroundings and ensure sustainable farming practices [13].

*Gibberella fujikuroi* produces gibberellic acid (GA3), a crucial signaling chemical, plant hormone, and growth regulator [14–16]. It has been observed to improve various physiological and biochemical processes in plants, particularly in extreme environmental circumstances [17–19]. GA3 plays a significant role in seed germination, stem elongation, flower initiation, cell expansion, fruit development, net photosynthetic rate, carbohydrate metabolism, antioxidant defense, and regulation of water uptake.

Jasmonates, including MJ and jasmonic acid (JA), play vital roles in plant stress responses and growth regulation [20, 21]. They promote MDA accumulation and inhibit chelator release, mitigating salt stress [22]. MJ triggers additional protective mechanisms. JA, a lipid-derived hormone, regulates various biological processes and is crucial for plant responses to salinity [23]. It influences protein patterns in wounded leaves and enhances plant antioxidant activity [24].

Canola (*Brassica napus* L.) is an important crop primarily grown for its edible oil, renowned for its rich polyunsaturated fatty acids [25]. Additionally, its by-products boast high protein levels. Particularly in semi-arid regions dominated by cereal cultivation, canola is a promising alternative crop due to its efficient water use [26]. Nevertheless, the increasing global demand for vegetable oil poses a notable challenge to oilseed production, particularly in regions vulnerable to prolonged salinity induced by ongoing climate change [27].

That’s why the current study aimed to explore the impact of MJ and GA3 on canola plants cultivated under salinity stress. This study is covering the knowledge gap regarding combined use of GA3 and MJ to alleviate salinity stress. The novelty of the current study lies in the utilization of GA3 and MJ as amendments for the improvement of canola growth cultivated in salt-affected soil. It is hypothesized that the combined use of GA3 and MJ might potentially improve the growth of canola plants under salinity stress.

## Material and method

### Experimental site and design

A pot study was conducted in the experimental area of ResearchSolution (30°09’41.6"N 71°36’38.0” E). Random sampling was done for pre-experimental soil characterization. A total of 5 samples were collected from the soil, and a composite sample was made, which was used for analysis. The experimental design was a completely randomized design (CRD). The physiochemical characteristics of soil and irrigation water are provided in Table 1.

**Table 1:** Pre-experimental soil and irrigation characteristics

Soil	Values	References	Irrigation	Values	References
pH	8.21	[28]	EC (µS/cm)	615	[29]
EC*e* (dS/m)	6.19	[30]	pH	7.11	
SOM (%)	0.55	[31]	Bicarbonates (meq./L)	5.14	
TN (%)	0.003	[32]	Carbonates (meq./L)	0.00	
Available P (µg/g)	5.34	[33]	Ca + Mg (meq./L)	4.21	
Extractable Na (µg/g)	84	[34]	Chloride (meq./L)	0.01	
Extractable K (µg/g)	111	[35]	Sodium (mg/L)	115	
Texture	Loam	[36]			

### MJ application and GA3 application

For making MJ solution, 95% pure salt was purchased from a certified dealer of Sigma-Aldrich in Multan. The characteristics of salt include the product name 392707-5ML, product number 0000257713, batch number SHBP6057, reference number 39924-52-2, and CAS number C_13_H_20_O_3_. The molecular formula of this compound is C_1__3_H_20_O_3_, and it has a molecular weight of 224.30. Initially, a 10 mM stock solution was made in acetone. Once the salt was dissolved, further dilutions were made per the treatment plan using deionized water. For making 5 mg/L GA3, a commercial-grade 10% GA3 (CAS 77-06-5; state powder; molecular formula C_19_H_22_O_6_; EINECS No. 201-001-0) tablet was purchased. GA3 was directly dissolved in water for foliar application.

### Seed collection and priming

The canola seeds were obtained from a certified supplier in Punjab, Pakistan. A sterilization procedure was followed, including sodium hypochlorite, ethanol, and deionized water. 20 seeds were planted in pots with 5 kg of soil, and after germination, a thinning process was used to maintain 10 seedlings per pot.

### Treatment plan

There were 4 levels of MJ, i.e., control, 0.8, 1.6, and 3.2 mM MJ, which were applied as foliar with and without 5 mg/L GA3. A total of 3 foliar applications (200 ml per pot) of treatments with four replicates were made using deionized and sterilized water at 21, 35, and 49 days after germination. The treatments with four replicates include control, 0.8mM MJ, 1.6mM MJ, 3.2mM MJ, 5 mg/L GA3, 0.8mM MJ + 5 mg/L GA3, 1.6 mM MJ + 5 mg/L GA3, and 3.2mM MJ + 5 mg/L GA3.

### Fertilizer

For the cultivation of canola, essential macronutrients N, P, and K were applied in the form of calcium ammonium nitrate (CAN), single superphosphate (SSP), and sulfate of potash (SOP). N, P, and K application rates were 30, 20, and 25 kg/acre per pot, 0.56, 0.38, and 0.37 g/pot (15 kg soil).

### Irrigation

The management of irrigation for each pot was carefully executed by utilizing a moisture gauge (ADVANCED™; 4 in 1 Soil Meter; China). Diligent surveillance was conducted to guarantee wetness on the scale of ∼70% of the soil’s field capacity.

### Harvesting and data collection

Harvesting was done after 120 days of cultivation. The growth attributes, i.e., shoot length, were measured soon after harvesting using a meter rod. For dry weight measurement, samples were oven-dried at 65 °C ± 5 °C, and then readings were taken on weight balance.

### Chlorophyll and carotenoids content

Arnon’s standard protocol was followed to assess chlorophyll a, chlorophyll b, and total chlorophyll levels in freshly harvested wheat leaves using an 80% acetone solution [37]. The final absorbance was taken at 663 nm, 645 nm, and 470 nm.

$${\rm{Chlorophyll}\, {a}}\left( {\frac{{{\rm{mg}}}}{{\rm{g}}}} \right) = \frac{{\left( {12.7 \times {\rm{A}}663} \right) - \left( {2.69 \times {\rm{A}}645} \right) \times {\rm{V}}}}{{1000 \times {\rm{W}}}}$$

$${\rm{Chlorophyll}\, {b}}\left( {\frac{{{\rm{mg}}}}{{\rm{g}}}} \right) = \frac{{\left( {22.9 \times {\rm{A}}645} \right) - \left( {4.68 \times {\rm{A}}663} \right) \times {\rm{V}}}}{{1000 \times {\rm{W}}}}$$

$${\rm{Total}}\,{\rm{Chlorophyll}}\left( {\frac{{{\rm{mg}}}}{{\rm{g}}}} \right) = \frac{{20.2\left( {{\rm{A}}645} \right) + 8.02\left( {{\rm{A}}663} \right) \times {\rm{V}}}}{{1000 \times {\rm{W}}}}$$

$${\rm{Carotenoids}}\left( {\frac{{{\rm{mg}}}}{{\rm{g}}}} \right) = {\rm{OD}}480 + 0.114\left( {{\rm{OD}}\, 663} \right) - 0.638\left( {{\rm{OD}}\, 645} \right)$$

### Antioxidants

We assessed superoxide dismutase (SOD) activity by quantifying the reduction of nitro blue tetrazolium (NBT) at a wavelength of 560 nm [38]. A standard protocol was used to analyze POD activity at 420 nm. Assessment of CAT activity due to H_2_O_2_ decomposition by measuring the absorbance at 240 nm [39]. For APX activity, ascorbate oxidation was recorded in the presence of H_2_O_2_ at 290 nm [40]. Samples were extracted to assess MDA by reacting them with thiobarbituric acid (TBA). The final absorbance was measured at 532 nm [41]. The glutathione reductase (GR) activity was measured at 340 nm [42]. For ascorbate (AsA), 10% trichloroacetic acid was used. The final absorbance was taken at 525 nm [43]. Free proline was quantified using sulfosalicylic acid, glacial acetic acid, and ninhydrin solutions. The absorbance was measured at 520 nm [44].

### Nutrients analysis

For the analysis of nutrients in the plant samples, 2 types of digestion were performed; the first was with sulfuric acid, in which a digestion mixture was used [29]. The second one was done with the di-acid mixture to analyze P, K, Na, and Cl [45]. The standard protocols were followed to analyze N on Kjeldahl’s distillation apparatus, P on a spectrophotometer, K and Na on a flame photometer, and via titration me.

### Statistical analysis

The collected data were subjected to standard statistical analysis [46]. The mean comparison was performed using appropriate statistical tests (Fisher’s LSD), and significance was considered at *p* < 0.05 using OriginPro 2021 [47]. Paired comparisons and cluster plots were also made using OriginPro 2021.

## Results

### Shoot length and dry weight, no. of leaves/plant and flowering branches/plant

**Fig. 1:**
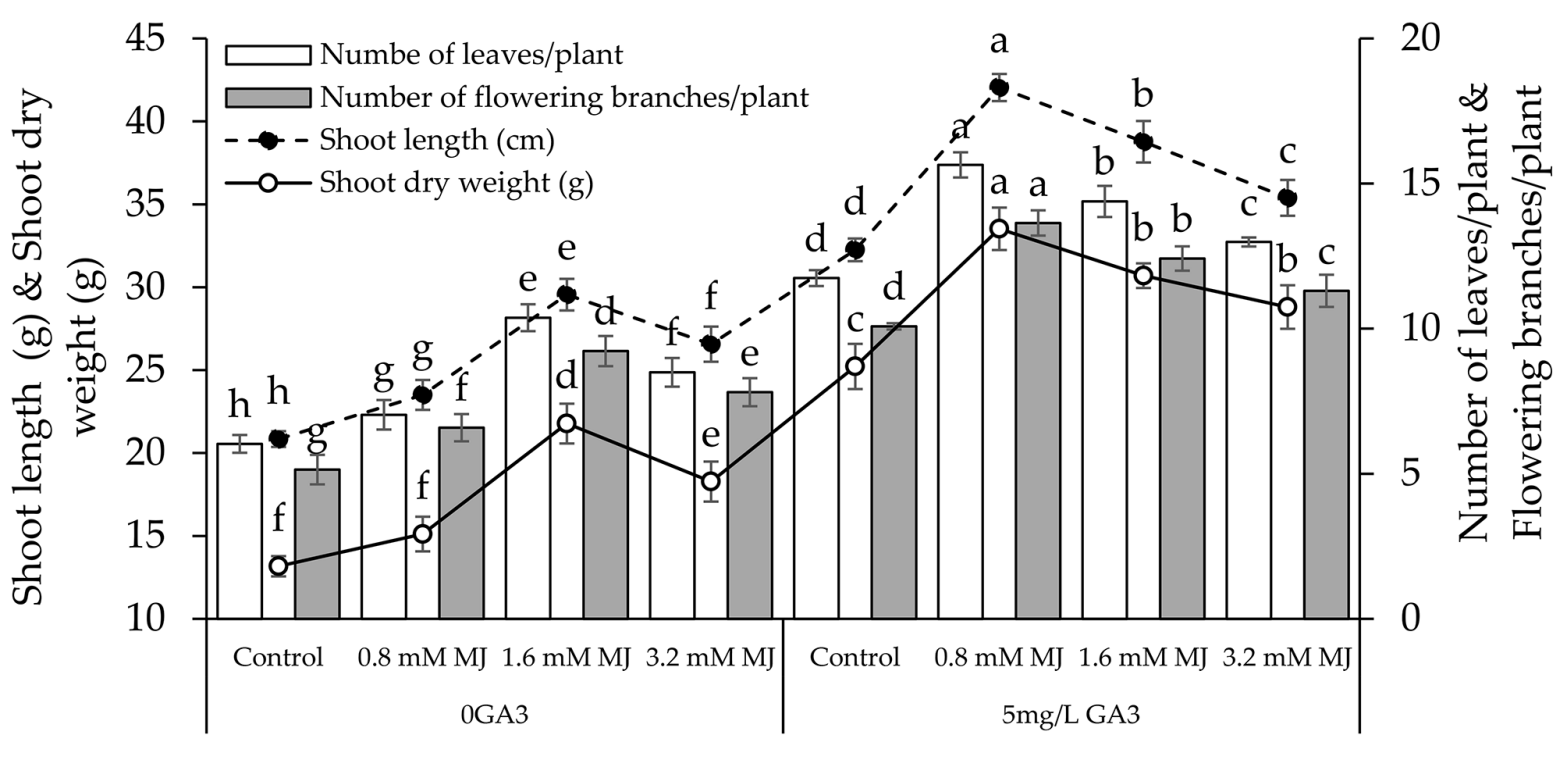
Effect of different levels of methyl jasmonate concentrations on the shoot length, shoot fresh weight, number of leaves/plant, and number of flowering branches/plant of canola grown under 0GA3 and 5mh/L GA3

Applying 0.8 mM MJ, 1.6 mM MJ, and 3.2 mM MJ with 0GA3 led to a notable increase in shoot length (11.33%, 41.79%, and 27.46%), shoot dry weight (12.85%, 65.33%, and 38.75%), no. of leaves/plant (14.26%, 72.10%, and 40.88%), and flowering branches/plant (21.94%, 79.44%, and 51.87%) than the control. Applying 0.8 mM MJ, 1.6 mM MJ, and 3.2 mM MJ with 5 mg/L GA3 showed a notable increase in shoot length (23.29%, 20.22%, and 9.72%), shoot dry weight (24.77%, 21.71%, and 14.21%), no. of leaves/plant (24.93%, 22.50%, and 10.60%), and flowering branches/plant (26.11%, 23.19%, and 12.15%) over the control (Fig. 1).

### Number of siliques per plant, seed yield and oil

**Fig. 2:**
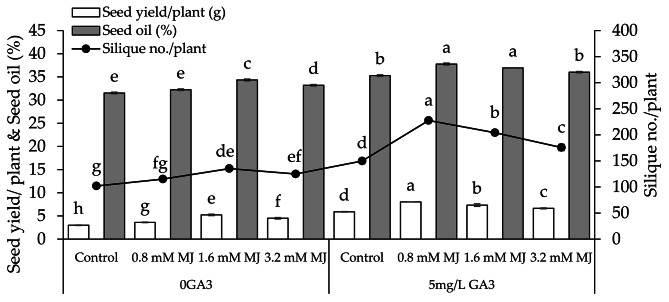
Effect of different levels of methyl jasmonate concentrations on the number of silique/plant, seed yield/plant, and seed oil of canola grown under 0GA3 and 5 mg/L GA3

With 0GA3, adding 0.8 mM MJ, 1.6 mM MJ, and 3.2 mM MJ treatments led to a notable increase in number of siliques per plant (11.52%, 32.80%, and 22.60%), seed yield (17.01%, 74.17%, and 50.08%), and seed oil (2.16%, 8.90%, and 5.30%) than the control. With 5 mg/L GA3, adding 0.8 mM MJ, 1.6 mM MJ, and 3.2 mM MJ treatments exhibit a notable rise in number of siliques per plant (34.16%, 36.23%, and 17.30%), seed yield (26.65% 24.58%, and 12.78%), and seed oil (6.62%, 4.72%, and 2.13%) from the control (Fig. 2).

### Chlorophyll and carotenoids content

**Fig. 3:**
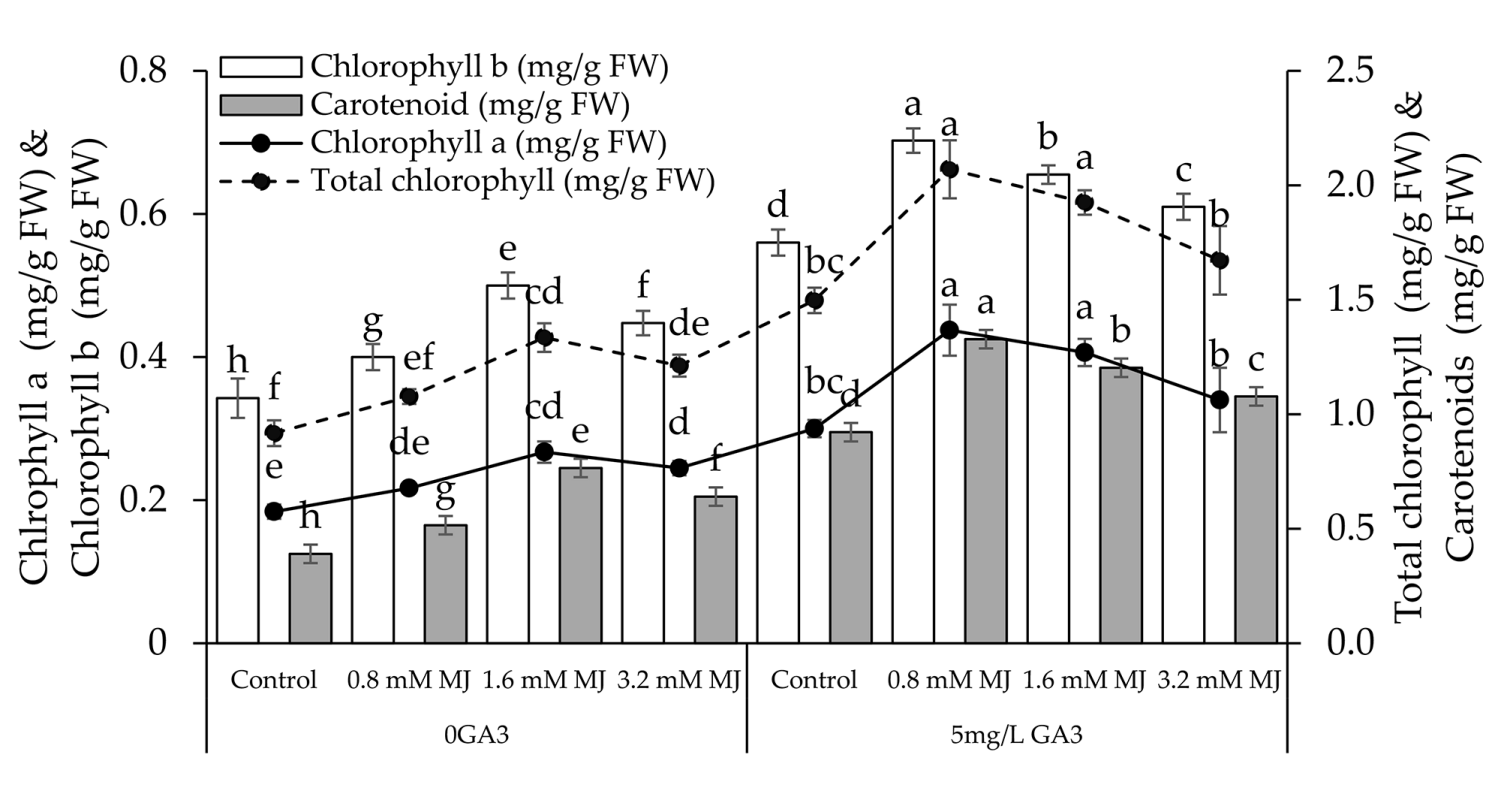
Effect of different levels of methyl jasmonate concentrations on the number of chlorophyll **a**, chlorophyll **b**, total chlorophyll, and carotenoids of canola grown under 0GA3 and 5 mg/L GA3

In the 0GA3, adding 0.8 mM MJ, 1.6 mM MJ, and 3.2 mM MJ treatments showed a significant rise in chlorophyll a (15.13%, 45.22%, and 33.04%), chlorophyll b (14.38%, 45.99%, and 30.66%), total chlorophyll (14.85%, 45.50%, and 32.15%), and carotenoids (24.24%, 96.00%, and 64.00%) compared to the control. These 0.8 mM MJ, 1.6 mM MJ, and 3.2 mM MJ treatments showed an improvement in chlorophyll a (31.44%, 35.47%, and 13.33%), chlorophyll b (20.28%, 16.96%, and 8.93%), total chlorophyll (27.66%, 28.55%, and 11.69%), and carotenoids (30.59%, 30.51%, and 16.95%) over the control under 5 mg/L GA3 (Fig. 3).

### Shoot and root proline, shoot and root total soluble carbohydrates

**Fig. 4:**
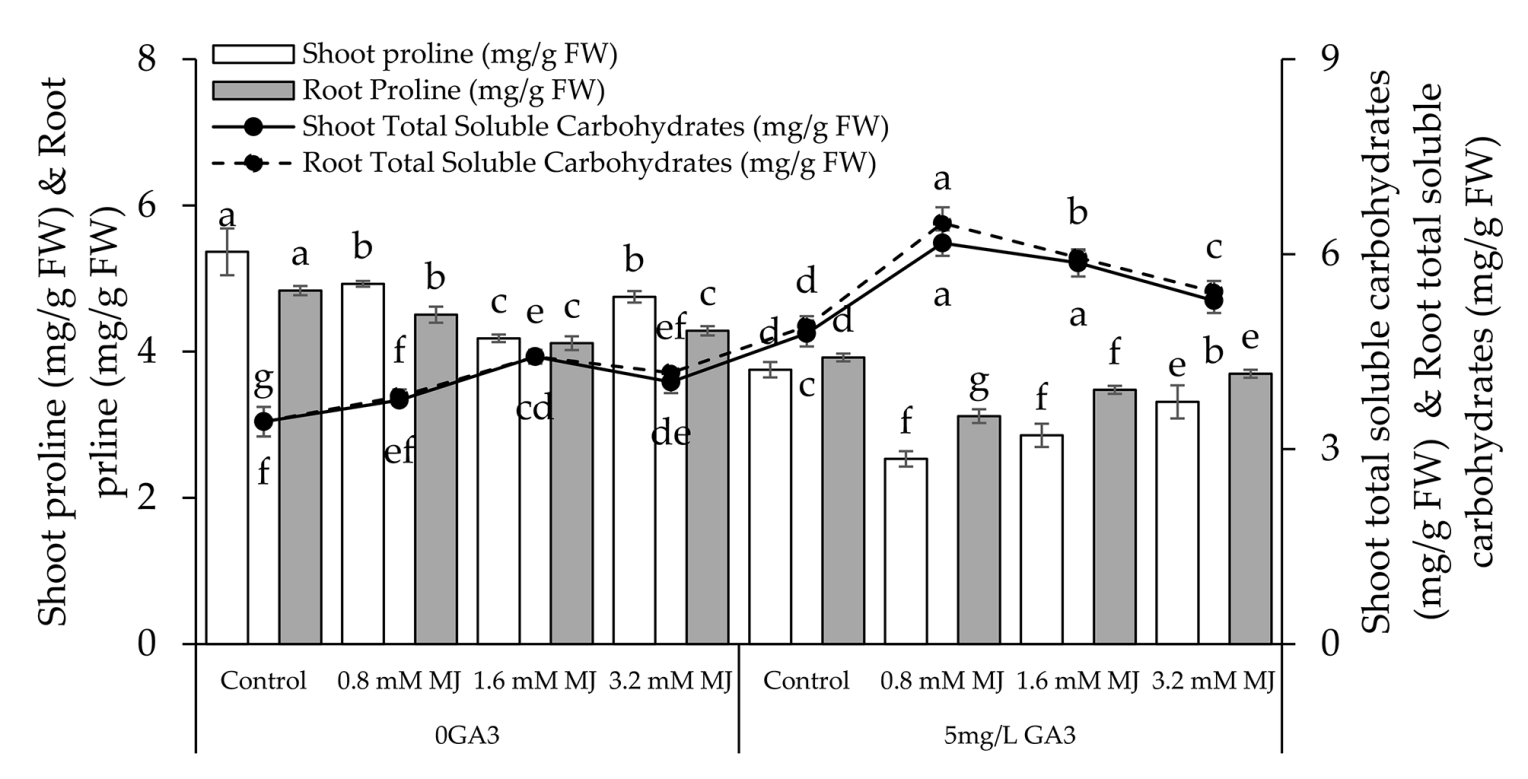
Effect of different levels of methyl jasmonate concentrations on the number of shoot proline, root proline, shoot total soluble carbohydrates, and root total soluble carbohydrates of canola grown under 0GA3 and 5 mg/L GA3

With 0GA3 application of 0.8 mM MJ, 1.6 mM MJ, and 3.2 mM MJ exhibit decreases in shoot proline (8.88%, 28.27%, and 12.95%) and root proline (7.33%, 17.50%, and 12.84%), and caused increase in shoot total soluble carbohydrates (8.79%, 29.22%, and 17.82%), and root total soluble carbohydrates (10.17%, 29.22%, and 21.99%) than the control. With 5 mg/L GA3 these 0.8 mM MJ, 1.6 mM MJ, and 3.2 mM MJ treatments showed decreases in shoot proline (48.17%, 31.44%, and 13.28%), root proline (25.74%, 12.72%, and 6.02%), and showed rise in shoot total soluble carbohydrates (22.53%, 22.70%, and 10.56%), and root total soluble carbohydrates (24.51%, 21.57%, and 10.89%) from the control (Fig. 4).

### Shoot and root ascorbic acid, shoot and root H_2_O_2_

**Fig. 5:**
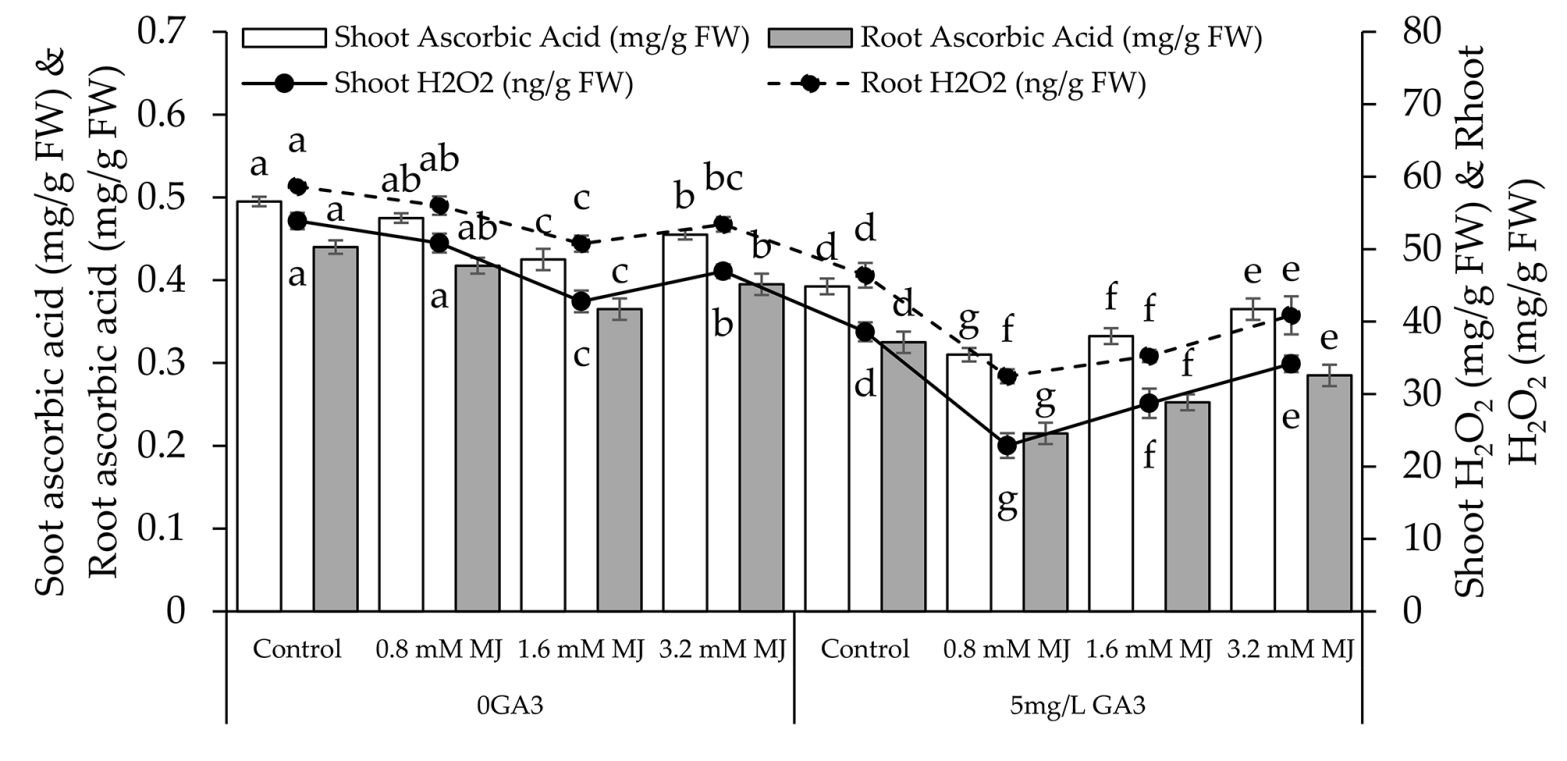
Effect of different levels of methyl jasmonate concentrations on the number of shoot ascorbic acid, root ascorbic acid, shoot H_2_O_2_, and root H_2_O_2_ of canola grown under 0GA3 and 5 mg/L GA3

A significant decrease in shoot ascorbic acid (4.21%, 16.47%, and 8.79%), root ascorbic acid (5.39%, 20.55%, and 11.39%), shoot H_2_O_2_ (6.01%, 25.97%, and 14.79%), and root H_2_O_2_ (4.70%, 15.54%, and 9.76%) was recorded with the application of 0.8 mM MJ, 1.6 mM MJ, and 3.2 mM MJ under 0GA3 over the control. Applying 0.8 mM MJ, 1.6 mM MJ, and 3.2 mM MJ treatments with 5 mg/L GA3 resulted in a significant decrease in shoot ascorbic acid (26.61%, 18.05%, and 7.53%), root ascorbic acid (51.16%, 28.71%, and 14.04%), shoot H_2_O_2_ (68.58%, 34.33%, and 12.91%), and root H_2_O_2_ (42.98%, 31.59%, and 13.53%) than the control (Fig. 5).

### Shoot and root MDA, shoot and root SOD

Under the 0GA3, applying 0.8 mM MJ, 1.6 mM MJ, and 3.2 mM MJ resulted decrease in shoot MDA (9.24%, 32.76%, and 20.11%), root MDA (8.10%, 32.22%, and 19.40%), shoot SOD (5.67%, 16.75%, and 10.73%), and root SOD (4.21%, 17.68%, and 9.60%) compared to the control. With the 5 mg/L GA3, applying 0.8 mM MJ, 1.6 mM MJ, and 3.2 mM MJ resulted decrease in shoot MDA (81.41%, 41.31%, and 18.94%), root MDA (99.91%, 43.33%, and 19.52%), shoot SOD (50.59%, 22.33%, and 11.64%), and root SOD (40.07%, 18.88%, and 10.76%) than the control (Table 2).

**Table 2:** Effect of different levels of methyl jasmonate concentrations on the shoot & root malondialdehyde (MDA), shoot and root superoxide dismutase (SOD) of canola grown under 0GA3 and 5 mg/L GA3

Treatment	Shoot MDA (µmol/g FW)	Root MDA (µmol/g FW)	Shoot SOD (U/mg FW)	Root SOD (U/mg FW)
	**0GA3**			
Control	15.75 ± 0.19a	17.45 ± 0.52a	85.39 ± 1.75a	68.78 ± 1.20a
0.8 mM MJ	14.42 ± 0.49b	16.14 ± 0.48b	80.81 ± 1.06b	66.00 ± 1.05b
1.6 mM MJ	11.87 ± 0.19d	13.20 ± 0.46d	73.14 ± 1.26d	58.45 ± 1.14d
3.2 mM MJ	13.12 ± 0.61c	14.62 ± 0.26c	77.12 ± 1.30c	62.76 ± 1.69c
	**5 mg/L GA3**			
Control	9.78 ± 0.78e	10.56 ± 0.82e	68.48 ± 2.54e	55.88 ± 0.69d
0.8 mM MJ	5.39 ± 0.38 h	5.28 ± 0.22 h	45.47 ± 4.75 h	39.89 ± 3.72 g
1.6 mM MJ	6.92 ± 0.05 g	7.37 ± 0.62 g	55.98 ± 1.22 g	47.00 ± 1.46f
3.2 mM MJ	8.23 ± 0.47f	8.83 ± 0.73f	61.34 ± 3.08f	50.45 ± 2.20e

The values are the mean of four replicates ± SE

### Shoot and root POD, shoot and root CAT

Adding 0.8 mM MJ, 1.6 mM MJ, and 3.2 mM MJ treatments with 0GA3 resulted significant decrease in shoot POD (3.70%, 12.97%, and 7.87%), root POD (5.22%, 12.14%, and 8.54%), shoot CAT (3.69%, 15.65%, and 10.63%), and root CAT (6.04%, 19.16%, and 12.01%) over the control. A significant decrease in shoot POD (14.81%, 10.15%, and 2.66%), root POD (21.08%, 8.71%, and 2.29%), shoot CAT (18.76%, 15.96, and 6.91%), and root CAT (29.95%, 17.88%, 7.87%) was observed with 0.8 mM MJ, 1.6 mM MJ, and 3.2 mM MJ treatments under 5 mg/L GA3 from the control (Table 3).

**Table 3:** Effect of different levels of methyl jasmonate concentrations on the shoot & root peroxidase (POD), shoot and root catalase (CAT) of canola grown under 0GA3 and 5 mg/L GA3

Treatment	Shoot POD (U/mg FW)	Root POD (U/mg FW)	Shoot CAT (U/mg FW)	Shoot CAT (U/mg FW)
	**0GA3**			
Control	55.89 ± 0.73a	59.28 ± 0.73a	28.00 ± 0.32a	29.25 ± 0.71a
0.8 mM MJ	53.90 ± 0.66b	56.34 ± 0.67b	27.01 ± 0.47b	27.59 ± 0.46b
1.6 mM MJ	49.48 ± 0.47d	52.87 ± 0.25d	24.21 ± 0.24d	24.55 ± 0.26d
3.2 mM MJ	51.81 ± 0.78c	54.62 ± 0.55c	25.31 ± 0.33c	26.12 ± 0.65c
	**5 mg/LGA3**			
Control	47.20 ± 0.42e	50.25 ± 0.53e	23.20 ± 0.62e	23.62 ± 0.61d
0.8 mM MJ	41.11 ± 0.55 h	41.51 ± 0.61 h	19.53 ± 0.44 g	18.17 ± 1.57 g
1.6 mM MJ	42.85 ± 1.05 g	46.23 ± 1.25 g	20.01 ± 0.09 g	20.03 ± 0.14f
3.2 mM MJ	45.98 ± 0.51f	49.13 ± 0.43f	21.70 ± 0.55f	21.89 ± 0.71e

The values are the mean of four replicates ± SE

### Shoot N, P, K, Na, and Cl

The use of 0.8, 1.6 and 3.2 mM MJ resulted in an increase of 4.35, 21.82 and 11.62% in shoot N respectively over control at 0GA3. Applying GA3 (5 mg/L) with 0.8, 1.6 and 3.2 mM MJ showed an enhancement of 10.38, 7.68, and 3.90% in shoot N respectively than control.

For shoot P, application of 0.8, 1.6 and 3.2 mM MJ resulted in 9.97, 28.14 and 18.56% enhancement compared to control under 0GA3. However, treatment GA3 (5 mg/L) caused an improvement of 15.22, 12.82, and 7.69% in shoot P than control.

In case of shoot K, 9.86, 32.45 and 20.39% enhancement were noted where 0.8, 1.6 and 3.2 mM MJ were applied respectively at 0GA3 over control. Furthermore, addition of 0.8, 1.6 and 3.2 mM MJ showed 8.05, 6.67, and 2.04% improvement in shoot K when applied with GA3 (5 mg/L) compared to control.

Results showed that shoot Na was 9.86, 31.07 and 20.19% decreased in 0.8, 1.6 and 3.2 mM MJ respectively at 0GA3. Similar kind of decline in shoot Na (47.46%, 25.22%, and 11.95%) was also noted when 0.8, 1.6 and 3.2 mM MJ were applied with GA3 (5 mg/L) over to control.

Regarding shoot Cl, a decline of 10.45, 33.46 and 20.26% was observed in 0.8, 1.6 and 3.2 mM MJ respectively than control under 0GA3. At 5 mg/L GA3, treatments 0.8, 1.6 and 3.2 mM MJ caused decrease, i.e., 91.14, 30.94, and 12.31% in shoot Cl compared to control respectively (Table 4).

**Table 4:** Effect of different levels of methyl jasmonate concentrations on the shoot N, P, K, Na, and Cl concentration of canola grown under 0GA3 and 5 mg/L GA3

Treatment	Shoot N (%)	Shoot P (%)	Shoot K (%)	Shoot Na (%)	Shoot Cl (%)
	**0GA**				
Control	1.59 ± 0.02 h	0.42 ± 0.02 h	1.80 ± 0.08f	2.17 ± 0.10a	107.48 ± 2.38a
0.8 mM MJ	1.66 ± 0.04 g	0.46 ± 0.01 g	2.04 ± 0.07e	1.98 ± 0.07b	97.31 ± 3.64b
1.6 mM MJ	1.94 ± 0.05e	0.54 ± 0.01e	2.39 ± 0.08c	1.66 ± 0.05d	80.53 ± 2.90d
3.2 mM MJ	1.78 ± 0.04f	0.50 ± 0.01f	2.17 ± 0.03d	1.81 ± 0.08c	89.37 ± 2.18c
	**5 mg/LGA3**				
Control	2.05 ± 0.04d	0.59 ± 0.01d	2.51 ± 0.02b	1.45 ± 0.09e	71.48 ± 1.91e
0.8 mM MJ	2.29 ± 0.02a	0.69 ± 0.01a	2.73 ± 0.01a	0.99 ± 0.08 h	37.40 ± 6.08 h
1.6 mM MJ	2.21 ± 0.03b	0.66 ± 0.01b	2.68 ± 0.05a	1.16 ± 0.06 g	54.59 ± 5.49 g
3.2 mM MJ	2.13 ± 0.02c	0.63 ± 0.01c	2.56 ± 0.04b	1.30 ± 0.05f	63.65 ± 4.07f

The values are the mean of four replicates ± SE

### Root N, P, K, Na, and Cl

Applying 0.8, 1.6 and 3.2 mM MJ resulted in an enhancement of 8.44, 27.39, and 18.60% in root N respectively over control at 0GA3. Applying 0.8, 1.6 and 3.2 mM MJ with GA3 (5 mg/L) showed an improvement of 15.82, 13.53, and 6.64% in root N respectively compared to control.

For root P, treatments 0.8, 1.6 and 3.2 mM MJ without GA3 caused 7.98, 26.59, and 19.36% increment compared to control under 0GA3. However, GA3 (5 mg/L) with 0.8, 1.6 and 3.2 mM MJ caused an increase of 12.73, 9.44, and 6.01% in root P than control.

In case of root K, 7.94, 20.03, and 16.71% enhancement were noted where 0.8, 1.6 and 3.2 mM MJ were applied respectively at 0GA3 over control. Furthermore, addition of 0.8, 1.6 and 3.2 mM MJ showed 13.27, 9.70, and 4.75%% improvement in root K when applied with GA3 (5 mg/L) compared to control.

Results showed that root Na was 5.73, 32.46, and 19.74% decreased in 0.8, 1.6 and 3.2 mM MJ respectively at 0GA3. Similar kind of decline in root Na (70.71, 53.27, and 11.72%) was also noted when 0.8, 1.6 and 3.2 mM MJ were applied with GA3 (5 mg/L) over to control.

Regarding root Cl, a decline of 13.59, 59.07, and 25.14% was observed in 0.8, 1.6 and 3.2 mM MJ respectively than control under 0GA3. At 5 mg/L GA3, treatments 0.8, 1.6 and 3.2 mM MJ caused decrease, i.e., 77.35, 29.59, and 10.21% in root Cl compared to control respectively (Table 5).

**Table 5:** Effect of different levels of methyl jasmonate concentrations on the root N, P, K, Na, and Cl concentration of canola grown under 0GA3 and 5 mg/L GA3

Treatment	Root N (%)	Root P (%)	Root K (%)	Root Na (%)	Root Cl (%)
	**0GA3**				
Control	1.46 ± 0.02 h	0.43 ± 0.01 h	1.88 ± 0.02 g	3.28 ± 0.08a	8.44 ± 0.47a
0.8 mM MJ	1.60 ± 0.03 g	0.47 ± 0.01 g	2.05 ± 0.09f	3.10 ± 0.13b	7.43 ± 0.25b
1.6 mM MJ	1.87 ± 0.04e	0.55 ± 0.01e	2.26 ± 0.03e	2.47 ± 0.05d	5.31 ± 0.10d
3.2 mM MJ	1.74 ± 0.07f	0.52 ± 0.00f	2.20 ± 0.02e	2.74 ± 0.10c	6.75 ± 0.42c
	**5 mg/LGA3**				
Control	2.00 ± 0.07d	0.58 ± 0.02d	1.88 ± 0.02 g	2.29 ± 0.09e	4.99 ± 0.09de
0.8 mM MJ	2.37 ± 0.02a	0.67 ± 0.01a	2.05 ± 0.09f	1.34 ± 0.12 h	2.82 ± 0.49 g
1.6 mM MJ	2.26 ± 0.05b	0.64 ± 0.01b	2.26 ± 0.03e	1.49 ± 0.02 g	3.85 ± 0.31f
3.2 mM MJ	2.13 ± 0.07c	0.62 ± 0.01c	2.20 ± 0.02e	2.05 ± 0.10f	4.53 ± 0.29e

The values are the mean of four replicates ± SE

#### Convex hull and hierarchical cluster analysis

**Fig. 6:**
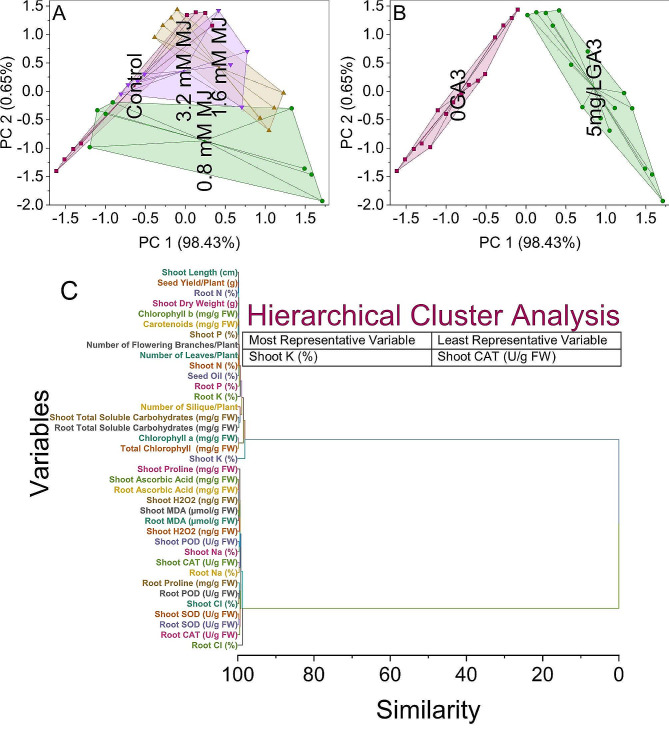
Cluster plot convex hull for treatments (**A**), GA3 levels (**B**), and hierarchical cluster plot (**C**) for studied attributes

In the PCA plot, the control group appears to cluster with negative values on both PC1 and PC2, indicating similar patterns in the measured variables. On the other hand, the group treated with 0.8 mM MJ is spread across the plot, with varying scores along both PC1 and PC2. This dispersion may suggest a more diverse response to the 0.8 mM MJ application, reflecting the individual variability within this group. Notably, the 0.8 mM MJ-treated samples tend to have negative scores on PC1, suggesting a commonality in their response, while PC2 captures additional variability. The samples that were treated with 1.6 mM MJ showed in separate clusters. The application of 1.6 mM MJ increases a particular reaction that sets it apart from both the control group and the group treated with 0.8 mM MJ (Fig. 6A).

We observed a clear distinction among the samples in the PCA plot based on their treatment conditions. Those treated with 0GA3, which served as the control, showed a tight clustering with negative scores on both PC1 and PC2. This clustering pattern indicates a similarity in how these samples responded to the absence of gibberellic acid (GA3). It suggests that without GA3, there’s a specific set of responses across the measured variables. Conversely, samples treated with 5 mg/L GA3 formed a separate cluster characterized by positive values on both PC1 and PC2. This clustering pattern suggests that applying 5 mg/L GA3 induced a response pattern distinct from the control group. The separation observed along PC2 indicates additional variability in the response to GA3 application, hinting at potentially diverse effects within this group. The PCA results reveal that GA3, specifically at the concentration of 5 mg/L, induces a distinct response pattern compared to the control condition (Fig. 6B).

Chlorophyll b and carotenoids share a similarity of 0.08911, suggesting a commonality in their response patterns. Similarly, shoot P and the combination of shoot ascorbic acid and root ascorbic acid cluster with similarities of 0.0946 and 0.13818, respectively. Variables such as seed yield/plant and root N show a similarity of 0.14592, indicating a shared response pattern. Additionally, shoot length and the combination of shoot MDA and root MDA clusters have similarities of 0.17572 and 0.17602, respectively. Further analysis reveals that variables related to oxidative stress, such as shoot H_2_O_2_, seed oil, and root POD, exhibit distinct clusters with varying similarities (Fig. 6C).

### Pearson correlation analysis

**Fig. 7:**
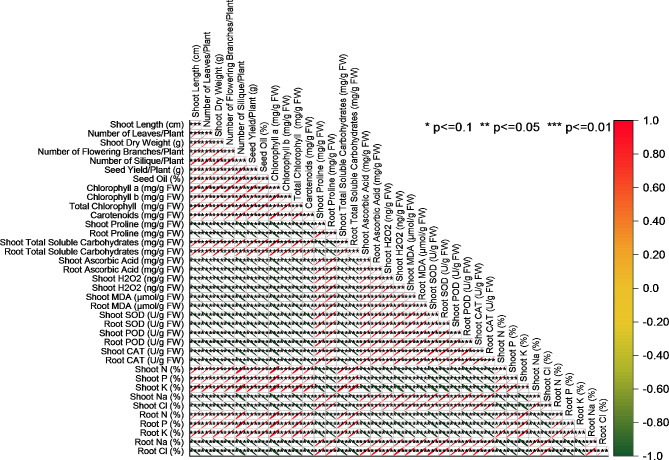
Pearson correlation for the studied attributes

shoot length displays strong positive correlations with several factors, including the number of leaves per plant (*r* = 0.99644), shoot dry weight (*r* = 0.99417), number of flowering branches per plant (*r* = 0.99483), seed yield per plant (*r* = 0.99671), seed oil content (*r* = 0.99396), total chlorophyll (*r* = 0.98485), and carotenoid content (*r* = 0.99806). Additionally, the number of leaves per plant exhibits high positive correlations with shoot dry weight (*r* = 0.99609), seed yield per plant (*r* = 0.99578), seed oil content (*r* = 0.99615), and carotenoid content (*r* = 0.99686), among others. Conversely, shoot length demonstrates strong negative correlations with shoot proline content (*r* = -0.98995), as does the number of leaves per plant (*r* = -0.98903) and shoot dry weight (*r* = -0.98892). Other notable negative correlations include shoot length with shoot ascorbic acid content (*r* = -0.99598) and root ascorbic acid content (*r* = -0.99521) (Fig. 7).

## Discussion

This study aimed to show the effects of MJ and GA3 applications on canola plants grown in salt-affected soils. These treatments were selected based on their potential to influence plant growth and physiological responses. They specifically focused on parameters such as shoot length, dry weight, leaf number, flowering branches, siliques per plant, seed yield, oil content, chlorophyll levels, and various biochemical attributes. The primary objective was to offer meaningful insights into optimizing canola cultivation in challenging salt-affected soil conditions through innovative hormonal applications. Compared to the control group, this study’s comprehensive findings reveal distinctive trends when applying GA3 and MJ applications to salt-affected soil [48]. Notable findings include enhanced shoot length, dry weight, and flowering attributes with specific concentrations of MJ under both 0 GA3 and 5 mg/L GA3 applications. Additionally, physiological parameters like chlorophyll content, proline, soluble carbohydrates, antioxidants, and nutrient uptake displayed significant variations, providing insights into the adaptive mechanisms employed by canola plants under salt stress in response to MJ and GA3 treatments. The observed trends resonate with established literature, supporting that hormonal applications can play a pivotal role in influencing various aspects of plant growth and development in challenging environmental conditions [49].

Investigating the impact of MJ and GA3 on canola cultivated in salt-affected soils revealed significant outcomes [48, 50]. Salinity stress often challenges plant growth by inducing reactive oxygen species (ROS) production [51, 52]. The combined application of MJ [53, 54] and GA3 [55] proved beneficial, activating key enzymes such as POD, SOD, and CAT in both roots and shoots. These enzymes act as effective ROS scavengers, preventing oxidative damage. Moreover, MJ and GA3 influenced the proline synthesis pathway in roots and shoots, enhancing osmotic regulation.

Furthermore, these regulators boosted the ascorbic acid pathway in both the roots and the shoots, which added to a strong antioxidant defense system [56, 57]. Moreover, a significant correlation exists between GA3 and increasing plants’ antioxidant defense mechanisms. Plant cells face oxidative damage due to ROS formation caused by salt, which is successfully mitigated by GA3 treatment [58]. Important antioxidant enzymes, including SOD, CAT, and POD, are activated more by GA3. These enzymes protect plant cells from oxidative stress, scavenging reactive oxygen species and eventually enhancing stress tolerance [59]. In canola addressing salt stress, the study demonstrates the synergistic effect of MJ and GA3 in improving stress tolerance by regulating root and shoot proline levels and affecting the ascorbic acid pathway [60].

Applying 0.8 mM MJ considerably enhanced shoot length and dry weight in canola plants; however, the greatest improvement across measures was observed with the 1.6 mM MJ treatment. Additionally, each plant produced more leaves and flowering branches; under 5 mg/L GA3, the 0.8 mM MJ treatment consistently outperformed other concentrations [61]. MJ concentrations in GA3 also favorably associated with the number of siliques, seed production, and oil content, suggesting that MJ may be useful in improving canola reproductive characteristics. With MJ treatments, the amount of chlorophyll rose noticeably, indicating an improvement in photosynthetic efficiency. Under some circumstances, especially in the 0.8 mM MJ application under 5 mg/L GA3 application, proline concentration decreased while total soluble carbohydrates rose, indicating modified metabolic pathways in response to stress. The results also revealed varying reactions in parameters linked to antioxidants; greater MJ concentrations generally resulted in lower activity, indicating a carefully regulated regulatory system to avoid excessive oxidative damage [62].

Additionally, concentration-dependent differences were observed in nutrient absorption; under 5 mg/L GA3, the 0.8 mM MJ treatment consistently resulted in enhanced nutritional content, highlighting its potential involvement in promoting the absorption of nutrients. Proline content responded complexly, declining in certain circumstances, especially in the case of the 0.8 mM MJ treatment combined with 5 mg/L GA3. The total amount of soluble carbohydrates rose, indicating stress-related changes to metabolic pathways [63–65]. The findings showed different reactions in parameters associated with antioxidants. The declines in ascorbic acid levels, H_2_O_2_, and MDA concentration highlight a challenging balance between reactive oxygen species and antioxidant defense systems. The activities of antioxidant enzymes showed dose-dependent responses, with lower activity often occurring at higher MJ concentrations [66]. Nutrient uptake displayed concentration-dependent variations. The 0.8 mM MJ with 5 mg/L GA3 application consistently improved nutrient content, emphasizing its potential role in enhancing nutrient acquisition.

MJ’s role in regulating plant development, stress responses, and secondary metabolite production is responsible for the observed improvements in growth parameters [54, 67]. Researchers have reported that MJ enhances plant tolerance to abiotic stress by modulating various physiological processes, such as antioxidant defense mechanisms, hormone signaling, and nutrient uptake [68]. The dose-dependent responses may be linked to the biphasic nature of MJ effects, where low concentrations induce specific responses. In contrast, higher concentrations might trigger different pathways or result in phytotoxic effects [69]. The optimal performance of the 0.8 mM MJ under 5 mg/L GA3 and 1.6 mM MJ under 0 GA3 application suggests a threshold beyond which the positive effects diminish. The interaction with GA3 could have influenced the overall outcomes, as GA3 is known to regulate plant growth and development [70]. The synergistic or antagonistic effects of GA3 and MJ on specific pathways may contribute to the observed variations. The findings align with previous studies indicating the positive impact of MJ on plant growth, stress tolerance, and yield. For several crops, comparable dose-dependent effects and optimum concentrations have been documented. Researchers have also observed the collaboration between MJ and GA3 on growth and stress responses, emphasizing the importance of specific strategies for certain crops and stress situations. The observed changes in chlorophyll concentration, antioxidant activity, and nutrient intake support previous research on the role of MJ in improving photosynthesis, reducing oxidative stress, and affecting nutritional assimilation under challenging conditions [71].

## Conclusion

It is concluded that, under salinity stress, canola growth was considerably increased by treatment 0.80%mM MJ + 5 mg/L GA3. Increased levels of chlorophyll in leaves and nutrients in roots and shoots showed the ability of 0.80 MJ + 5 mg/L GA3 to alleviate the effects of salt stress. This combination shows increased cell membrane integrity by successfully regulating enzyme activities, including MDA, POD, SOD, APX, and CAT, against salt stress. Growers can apply 0.80%mM MJ + 5 mg/L GA3 to improve canola cultivation under salinity stress significantly. More investigations are also suggested at the field level to declare 0.80%mM MJ + 5 mg/L GA3 as the best amendment for alleviating salinity stress in canola plants in different climatic conditions.

## Data Availability

All data generated or analysed during this study are included in this published article.

## Declarations

### Experimental research and field studies on plants (either cultivated or wild), including the collection of plant material, must comply with relevant institutional, national, and international guidelines and legislation

We confirmed that all methods were performed in accordance with the relevant guidelines/regulations/ legislation.
